# Expression and Prognostic Value of Oct-4 in Astrocytic Brain Tumors

**DOI:** 10.1371/journal.pone.0169129

**Published:** 2016-12-28

**Authors:** Jeanette Krogh Petersen, Per Jensen, Mia Dahl Sørensen, Bjarne Winther Kristensen

**Affiliations:** 1 Department of Pathology, Odense University Hospital, Odense C, Denmark; 2 Department of Clinical Research, University of Southern Denmark, Odense C, Denmark; University of Portsmouth, UNITED KINGDOM

## Abstract

**Background:**

Glioblastomas are the most frequent type of malignant primary brain tumor with a median overall survival less than 15 months. Therapy resistance of glioblastomas has been attributed to the presence of tumor initiating stem-like cells (TSCs). TSC-related markers have therefore been suggested to have promising potentials as prognostic markers in gliomas.

**Methodology/Principal Findings:**

The aim of the present study was to investigate the expression and prognostic impact of the TSC-related marker Oct-4 in astrocytic brain tumors of increasing grade. In total 114 grade II, III and IV astrocytic brain tumors were immunohistochemically stained for Oct-4, and the fraction and intensity of Oct-4 positive cells were determined by morphometric analysis of full tumor sections. Oct-4 was expressed in all tumors, and the Oct-4 positive cell fraction increased with tumor grade (p = 0.045). There was no association between survival and Oct-4 positive cell fraction, neither when combining all tumor grades nor in analysis of individual grades. Oct-4 intensity was not associated with grade, but taking IDH1 status into account we found a tendency for high Oct-4 intensity to be associated with poor prognosis in anaplastic astrocytomas. Double immunofluorescence stainings showed co-localization in the perivascular niches of Oct-4 and two other TSC markers CD133 and nestin in glioblastomas. In some areas Oct-4 was expressed independently of CD133 and nestin.

**Conclusions:**

In conclusion, high Oct-4 fraction was associated with tumor malignancy, but seemed to be without independent prognostic influence in glioblastomas. Identification of a potential prognostic value in anaplastic astrocytomas requires additional studies using larger patient cohorts.

## Introduction

Astrocytic brain tumors are the most common type of gliomas and the most common type of primary central nervous system tumors. Based on histological appearance these tumors are graded into four World Health Organization (WHO) grades I-IV. The most frequent and malignant glioma is the glioblastoma (GBM), which is a WHO grade IV tumor. GBMs have a fatal course, with a median survival of 14.6 months from the time of diagnosis. After introducing the new standard treatment for GBM, consisting of maximal surgical resection, radiotherapy and concomitant and adjuvant chemotherapy with temozolomide, the overall survival has improved [1]. Robust and clinical useful biomarkers are therefore needed for GBM patients as well as patients with lower grade astrocytic tumors.

The presence of tumor initiating stem-like cells (TSCs) in glioblastomas [2] have been put forward as a major reason explaining resistance against radio- and chemotherapy [3]. TSCs are hypothesized to have capacity for self-renewal, symmetric and asymmetric cell division and endless proliferation thereby being responsible for tumor recurrence [4, 5]. TSCs have also been suggested to be present in cancers of the breast [6], colon [7], ovary [8], pancreas [9], prostate [10], lungs [11] and oral cancer [12].

Octamer-binding transcription factor 4 (Oct-4) has been suggested to be a TSC-related marker being expressed both in embryonic stem cells and germ cells, where it is responsible for stem cell pluripotency, self-renewal and regulation of differentiation [13]. Oct-4 is part of the POU family of transcription factors (POU5F1) [14]. The POU domain binds to DNA via a double helix-turn-helix structure thereby regulating messenger RNA transcription [15]. Oct-4 has been identified in several cancer types including lung cancer [11], bladder cancer [14], and oral cancer [12]. Previously, Oct-4 has been identified in gliomas and glioma cell lines by immunohistochemistry (IHC) and real-time PCR (RT-PCR). An increase in Oct-4 expression with tumor grade has been reported in a study with 41 astrocytic and oligodendroglial gliomas assessed by pathologist-based scoring [16]. No association with survival was found in a study comparing survival with Oct-4 IHC levels in tissue micro-arrays (TMAs) using pathologist-based scoring of astrocytic and oligodendroglial gliomas [17].

The aim of this study was to investigate the expression and prognostic potential of Oct-4 protein in 114 astrocytic WHO grade II-IV brain tumors. We were encouraged to perform this study by finding an association between increasing Oct-4 mRNA levels and grade as well as shorter overall survival in the TCGA dataset. In contrast to observer-dependent pathologist-based scoring, we counted the Oct-4 positive cells in full IHC stained sections using random systematic sampling (meander) and estimated labelling fractions and intensity levels. We have previously used this and similar approaches in other biomarker studies [4, 18, 19]. Possible co-expression of Oct-4 with two other TSC-related markers, CD133 and nestin was investigated by double immunofluorescence staining,—a methodology we have used in previous studies [4, 19, 20].

## Materials and Methods

### Patients

Archival tumor material was obtained from 114 patients who underwent initial surgery of diffuse astrocytoma (DA, N = 24) and anaplastic astrocytoma (AA, N = 18) between 1994–2005 and glioblastoma (GBM, N = 72) between 2001–2005 at Odense University Hospital, Denmark. The patients had received no prior treatment to craniotomy. Recurrent tumors, which are known to change the expression of a number of proteins were not included in the study as well as tumors removed exclusively by ultrasonic aspiration since this procedure is known to cause tissue degeneration [21]. The material has been used to investigate the prognostic potential of other markers [4, 22, 23]. To encompass a suitable area for quantification and expression assessment, biopsies with a diameter less than 4 mm were excluded.

### Ethics

The study was approved by the Regional Scientific Ethical Committee of the Region of Southern Denmark, which is the official Danish ethical review board in the Region of Southern Denmark. Since this study was a retrospective study with archival brain tumor tissue, no written or verbal consent should be obtained.

### Oct-4 immunohistochemistry

After the surgical removal, tumor tissue was fixed in 4% neutral buffered formaldehyde and embedded in paraffin. Paraffin sections of all tumors were stained using Oct-4 polyclonal rabbit antibody, clone 19857 (Abcam). The immunostainings were performed using a Dako Autostainer Universal Staining System (DAKO, Glostrup, Denmark). Paraffin sections of 3 μm were deparaffinised and endogenous peroxidase activity was quenched by immersion in 1.5% hydrogen peroxidase followed by heat-induced epitope retrieval (HIER) in T-EG buffer. The sections were subsequently incubated for 60 min with the primary antibody diluted (1:800) in antibody diluent (Dako, Denmark). The detection of the antigen-antibody complex was performed using Advanced HRP (Dako, Denmark) followed by visualisation with diaminobenzidine (DAB) as chromogen. Finally, the sections were counterstained using Mayer’s haematoxylin (Bie & Berntsen, Denmark). Paraffin sections of tissue microarrays with 28 normal human tissues and 12 human cancers were used as control tissue. Oct-4 was only found with intense and specific nucleus expression in seminoma. When performing the immunostainings, positive and negative controls were always included. Primary antibody omission was used as a negative control. Tissue sections from GBMs with different fixation times in 4% NBF (1h, 6h, 12h, 24h, and 48h) showed no differences in staining fraction or intensity.

### Quantification of Oct-4 staining

For quantification of the Oct-4 staining, the stereological analysis program, Computer Assisted Stereological Toolbox (CAST2, Olympus Inc., Denmark) software was used. After Oct-4 staining, the 114 tumors were blinded, numerated randomly and quantified. Mean Oct-4 positive tumor cell fractions were obtained by counting both positive and negative cells in systematic random fields for the whole tissue section. Above 200 Oct-4 positive tumor cells were counted for each tumor. For each tumor, tumor cell fraction was calculated by dividing the number of Oct-4 positive tumor cells with the number of total tumor cells. The staining intensity was calculated as a mean intensity value based on intensities for each of the systematic random fields; the score 0 corresponds to no staining, score 1 to weak staining, score 2 to moderate staining and score 3 to pronounced staining. Necrotic areas and areas with extensive bleeding were not included. The invasion zone, positive granulocytes, blood vessels as well as non-tumorous tissue were also excluded from the quantification.

### Oct-4 double immunofluorescence staining

Double immunofluorescence staining was performed on 3 μm paraffin sections from 10 GBMs. The sections were deparaffinised and endogenous peroxidase activity quenched followed by Heat-Introduced Epitope Retrieval, HIER. The stainings were performed using a DAKO Autostainer Universal Staining System. When performing CD133/Oct-4 double immunofluorescence stainings the tissue sections were first incubated with CD133 antibody (W6B3C1, Miltenyi Biotec, Germany) and CD133 detected by CSA II Biotin-free Tyramide Signal Amplification System (DAKO, Denmark). A second round of HIER with T-EG was performed, sections incubated with OCT-4 and detection performed by TSA Plus Fluorescein system (PerkinElmer, Denmark). The cover slips were mounted using Vector mounting medium with DAPI (Vector, Denmark). When performing the Nestin/Oct-4 double immunofluorescence staining, sections were first incubated with Oct-4 followed by detection with TSA Plus Fluorescein system (PerkinElmer, Denmark). After a second round of HIER, sections were incubated with nestin (196908, R&D Systems, UK) and detection performed with Alexa flour 488 Donkey Anti mouse (Invitrogen, Denmark). The cover slips were mounted as above. Co-expression was visualised using a Leica DM 50000B microscope with DAPI (Vysis), FITC and Rhodamine filters (Leica) at 400x magnification.

### IDH1 immunohistochemistry

In order to detect the most frequent IDH mutation, sections of all DA (N = 24) and most AA (N = 17) were stained with the mIDH1R132H antibody (mIDH1R132H, clone H14, Dionova, 1: 100) using the BenchMark Ultra IHC/ISH staining system (Ventana Medical Systems, Inc, AZ, USA) as previously described [24, 25].

### Patient dataset analysis

The Oct-4 mRNA expression (POU5F1) was explored using GlioVis (https://gliovis.bioinfo.cnio.es) and exported directly from the program. The results shown are based upon data generated by the The Cancer Genome Atlas (TCGA) Research Network: http://cancergenome.nih.gov/ [26, 27]. Data was available for 620 (grade) and 667 (survival) glioma patients. For the GBMs (N = 152), mRNA expression data for Ki67 (MKI67), CD133 (PROM1) and nestin (NES) were exported from GlioVis and correlated to the Oct-4 expression.

### Statistical analysis

For comparison of mean fractions of positive Oct-4 tumor cells and mean tumor cell staining intensities between the grades, 1-way ANOVA with Bonferroni correction was used. Correlation analyses were performed using Spearman’s rank correlation test. Student’s unpaired t-test was used for comparison of IDH-1 wildtype (wtIDH1) and IDH-1 mutated (mIDH1) tumors. Overall survival was defined from day of initial surgery until death of the patient. For all tumors and each tumor grade, the Oct-4 positive tumor cell fraction and mean staining intensity was compared with overall survival in Kaplan-Meier plots using the log rank test. The median was used as a predefined cut-off value. Additional cutpoint analyses revealed no better cutoff than the median. The multivariate Cox regression model was used to adjust for age and gender for patients with GBM and to adjust for IDH-1 status for patients with AA. All assumptions regarding Cox regression were tested, and all analyses were carried out in GraphPad (Prism) or STATA version 14 (StataCorp LP, Texas, USA) using an overall significance level of p<0.05.

## Results

### Oct-4 mRNA expression in the TCGA dataset

**Fig 1:**
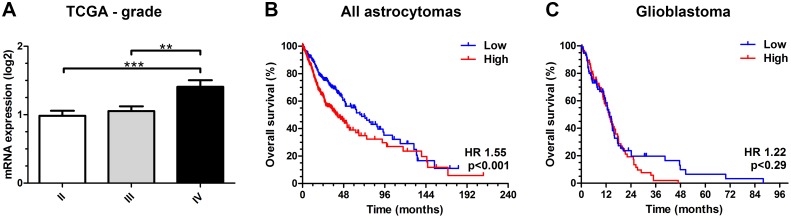
Oct-4 (POU5F1) mRNA expression in the TCGA dataset. (A) The mRNA levels were significantly higher in grade IV tumors compared to grade II (p<0.001) and grade III (p<0.01) tumors. (B) High expression was associated with shorter overall survival when all grades were combined (p<0.001), (C) but did not show any prognostic impact in patients with glioblastoma (p = 0.29).

To investigate the association between Oct-4 and tumor malignancy as well as patient prognosis in gliomas, we evaluated the mRNA expression of Oct-4 (POU5F1) in the TCGA dataset and found that patients with WHO grade IV tumors had higher Oct-4 expression than grade WHO II (p<0.001) and WHO III (p<0.01) tumors (Fig 1A). High Oct-4 predicted poorer survival (HR 1.55; 95% CI 1.20–2.01; p<0.001) when dichotomized at the median (Fig 1B). Oct-4 expression was not significantly associated with prognosis in GBMs dichotomized at the median (HR 1.22; 95% CI 0.84–1.77; p = 0.29) (Fig 1C). We also investigated potential correlation between mRNA levels of Oct-4, Ki-67, CD133 and nestin in the TCGA dataset. No significant correlation was found between Oct-4 mRNA expression level and Ki-67 (r_s_ = 0.02; p = 0.85), CD133 (r_s_ = 0.09; p = 0.27) and nestin (r_s_ = -0.03; p = 0.68) mRNA expression levels, respectively.

### Oct-4 expression in normal brain tissue and control tissue

**Fig 2:**
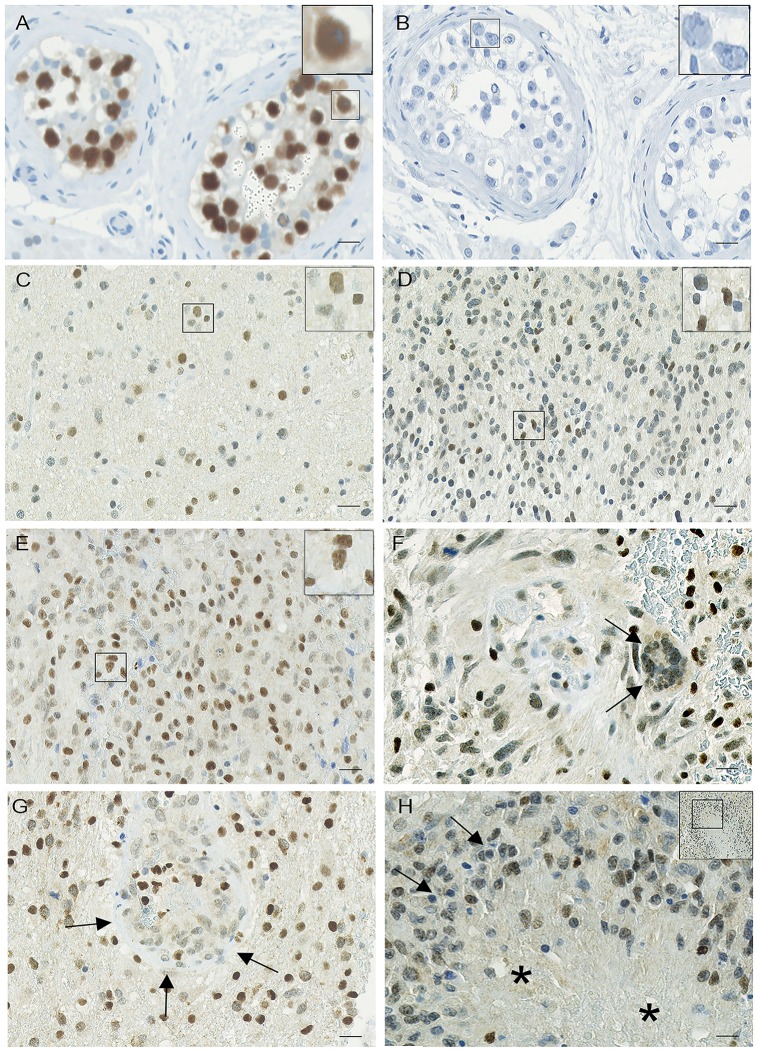
Oct-4 immunohistochemical expression in astrocytic brain tumors. (A) The positive seminoma tissue control showed an intense and specific nuclear staining. (B) Omission of the primary antibody abolished all staining reaction. Representative Oct-4 immunostaining with high expression levels in (C) diffuse astrocytomas, (D) anaplastic astrocytomas and (E) glioblastomas. (F) Multinuclear giant cell (arrows) and (G) glomeruloid tufts (arrows) in glioblastomas showed positive staining of a fraction of the cells. (H) Pseudopalisading cells (arrows) around necrosis (asterisk) without enhanced staining. Scale bar indicates (A-H) 50 μm, 400x.

In normal brain tissue, Oct-4 showed no staining of glial cells and neurons. A low staining intensity was seen in a small fraction of endothelial cells of the blood vessels. Weak to moderate staining was seen in cells in the subventricular zone. Positive staining was also seen in granulocytes. An intense and specific nuclear staining was observed only in seminoma (Fig 2A). Oct-4 was not detected in normal human adult testis tissue (Fig 2B).

### Oct-4 expression in astrocytic brain tumors

Patient characteristics and Oct-4 levels are shown in Table 1. Oct-4 positive tumor cells were observed in all 114 astrocytic brain tumors. The overall staining pattern was heterogeneous but at the cellular level the staining was predominantly localized in tumor nuclei, although weak cytoplasmic staining was also seen. The staining intensity varied considerably between samples and between different regions within the same sample. In DAs, both tumors with fibrillary and gemistocytic morphologies expressed Oct-4 with low, moderate and high expression levels, respectively (Fig 2C). AAs resembled DA in expression pattern, but had to some extent a more intense staining (Fig 2D). GBM tumor cells with various morphologies also expressed Oct-4 at different levels, but in general with a more specific intense nuclear staining compared with DAs and AAs (Fig 2E). The nuclei of multinuclear giant cells in GBM often showed various intensities of Oct-4 staining, most often weak to moderate staining intensities (Fig 2F, arrows). Besides being expressed in the tumor cells also glomeruloid tufts (Fig 2G, arrows) and other blood vessels showed positive staining. The staining did not appear to be enhanced in pseudopalisading cells (Fig 2H, arrows) around necrosis (Fig 2H, asterisk). Negative blood vessels were also seen, although less commonly.

**Table 1:** Patient characteristics and Oct-4 levels.

*Parameter*	*DA*	*AA*	*GBM*	*All astrocytomas*
***Patients (n)***	24	18	72	114
***Gender***				
*Male*	16 (67%)	11 (61%)	46 (64%)	73 (64%)
*Female*	8 (33%)	7 (39%)	26 (36%)	41 (36%)
***Age***				
*Mean*	44.9	54.0	61.3	56.7
*Range*	2.6–78.5	14.7–77.3	21.2–78.4	2.6–78.5
***Status (n)***				
*Alive*	5 (21%)	0 (0%)	1 (1%)	6 (5%)
*Dead*	19 (79%)	18 (100%)	71 (99%)	108 (95%)
***OS (months)***				
*Median*	56.3	18.4	8.4	10.6
*Range*	2.1–247.8	2.2–110.1	0.07–160.0	0.07–247.8
***Fraction***				
*Mean* ± *SD*	0.13 ± 0.083	0.14 ± 0.097	0.20 ± 0.16	0.18 ± 0.14
***Intensity***				
*Mean* ± *SD*	1.74 ±0.53	1.55 ± 0.50	1.81 ± 0.47	1.76 ± 0.49

**Abbreviations:** DA, diffuse astrocytoma; AA, anaplastic astrocytoma; GBM, glioblastoma multiforme; SD, standard deviation.

### Distribution of Oct-4 positive tumor cell fraction and intensity

**Fig 3:**
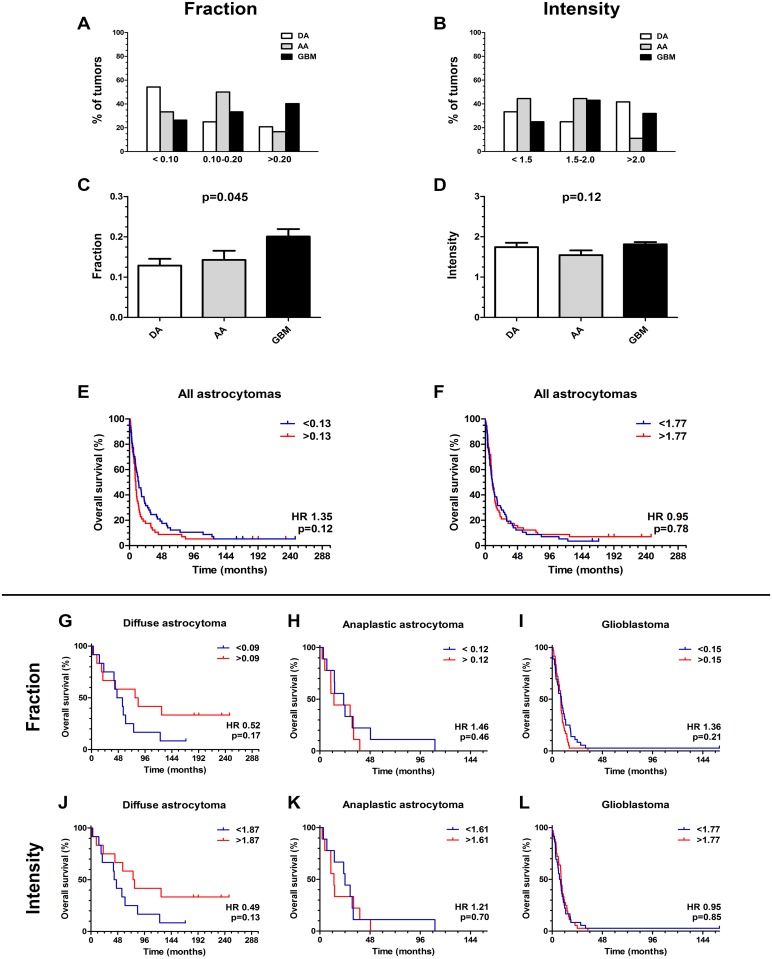
Distribution of Oct-4 staining and Kaplan Meier Survival analysis. For tumor cell fraction, glioblastomas were the most frequent tumors in the high expression group (>20%), whereas anaplastic astrocytomas were the most frequent tumors in the group with median expression level (10–20%). (A) Over 50% of diffuse astrocytomas was located in the low expression group (0–10%), where also the lowest percentage of glioblastomas was located. (B) For tumor cell staining intensity no clear pattern of distribution was found. (C) Comparing Oct-4 expression with grade there was a significant association of Oct-4 fraction with grade (p = 0.045), (D) whereas the Oct-4 intensity was similar for the individual grades (p = 0.12). (E-L) Kaplan-Meier plots for all astrocytomas showed no statistically significant association of Oct-4 (E) fraction and (F) intensity with overall survival. (G, J) For diffuse astrocytomas, (H, K) anaplastic astrocytomas and (I, L) glioblastomas no statistically significant association between Oct-4 fraction/intensity and overall survival was found. For comparison of grades, data are shown as means +SEM and ANOVA with Bonferroni correction was used for comparison of the different tumor grades.

GBMs were the most frequent tumors in the high expression group (>20%). In the group with 10–20% expression level AAs were most frequent, whereas DAs were most frequent in the low expression group (0–10%). The lowest percentage of GBMs was also located in this group (Fig 3A). Oct-4 staining intensity was almost found evenly distributed between the three groups and no clear pattern of distribution was found (Fig 3B). One-way ANOVAs showed that the Oct-4 fraction increased with WHO grade (p = 0.045), however in the postanalysis there was no significant differences between the individual pairings (Fig 3C). The Oct-4 intensity was similar for all grades (p = 0.12) (Fig 3D).

### Oct-4 expression and patient survival

**Fig 4:**
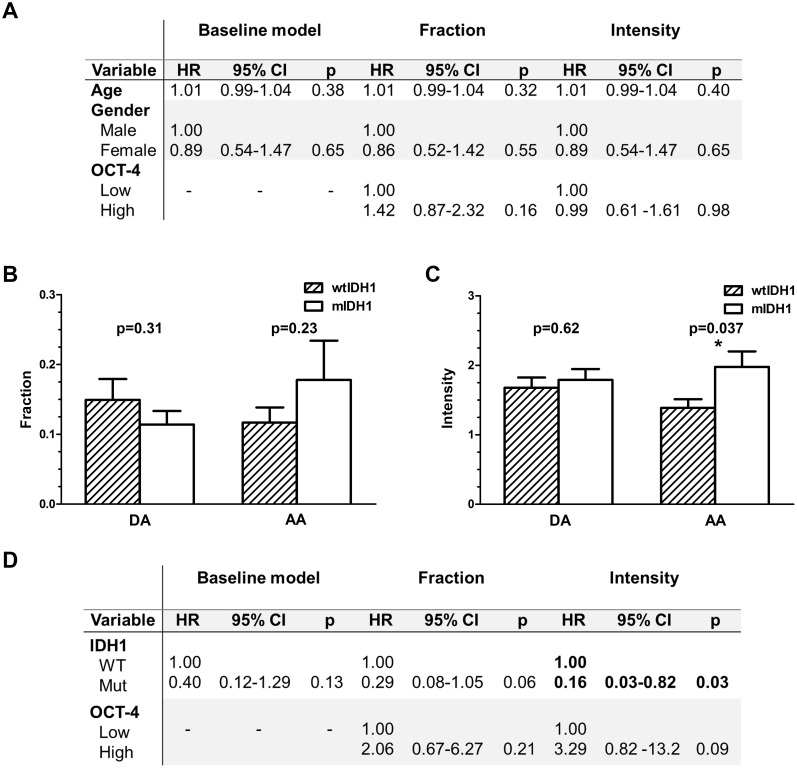
Multivariate analysis and association between Oct-4 and IDH1 status. (A) Oct-4 did not have any independent prognostic value in glioblastomas when gender and age were taken into account. (B) No significant difference in Oct-4 fraction was found for IDH-1 wildtype (wtIDH1) and IDH-1 mutated (mIDH1) tumors, neither for diffuse astrocytomas (p = 0.31) nor for anaplastic astrocytomas (p = 0.23). (C) For Oct-4 intensity the same results were obtained in diffuse astrocytomas (p = 0.62), while for anaplastic astrocytomas mIDH1 tumors showed higher intensity than wtIDH1 tumors (p = 0.037). (D) Performing multivariate analysis on patients with anaplastic astrocytomas, mIDH1predicted improved survival (p = 0.03), while high Oct-4 tended to be associated with poor prognosis (p = 0.09).

Kaplan-Meier plots for all astrocytomas illustrating Oct-4 tumor cell fraction showed a weak tendency for an association of high Oct-4 with shorter overall survival (Fig 3E). Oct-4 intensity did not seem to influence overall survival of all astrocytomas (Fig 3F). For patients with DA, fraction (HR 0.52; 95% CI 0.20–1.33; p = 0.17) and intensity (HR 0.49; 95% CI 0.19–1.23; p = 0.13) was not significantly associated with overall survival (Fig 3G and 3J). Similar for AAs (Fig 3H and 3K) and GBMs (Fig 3I and 3L) we did not find any significant association between overall survival and Oct-4 fraction (AA: HR 1.46; 95% CI 0.54–3.94; p = 0.46 and GBM: HR 1.36; 95% CI 0.84–2.21;p = 0.21) or intensity (AA: HR 1.21; 95% CI 0.46–3.19; p = 0.70 and GBM: HR 0.95; 95% CI 0.60–1.52; p = 0.85). The multivariate Cox regression test adjusting for age and gender revealed that the tumor cell fraction and intensity in GBMs did not influence the overall patient survival independently (Fig 4A).

### Oct-4 and IDH1 status in diffuse and anaplastic astrocytomas

IDH1 status was determined for all DAs (10 wtIDH1 and 14 mIDH1) and 17 of the 18 AAs (13 wtIDH1 and 4 mIDH1). No difference between mIDH1 and wtIDH1 was observed for Oct-4 fraction in either DAs (p = 0.31) or AAs (p = 0.23) (Fig 4B). Oct-4 intensity did not differ between mIDH1 and wtIDH1 in DAs (p = 0.62), but the intensity was significantly higher in mIDH1 AAs compared to wtIDH1 AAs (p = 0.037) (Fig 4C). Multivariate analysis including Oct-4 expression and IDH1 status was therefore performed on patients with AAs (Fig 4D). IDH1 status in the baseline model did not reach significance, however, when accounting for Oct-4 fraction and intensity, mIDH1 tended as expected to associate or significantly associate with better prognosis (p = 0.06 and p = 0.03, respectively). High Oct-4 levels, especially high intensity, tended to predict a shorter patient survival (p = 0.21 and p = 0.09).

### Co-expression of Oct-4 with CD133 and nestin

**Fig 5:**
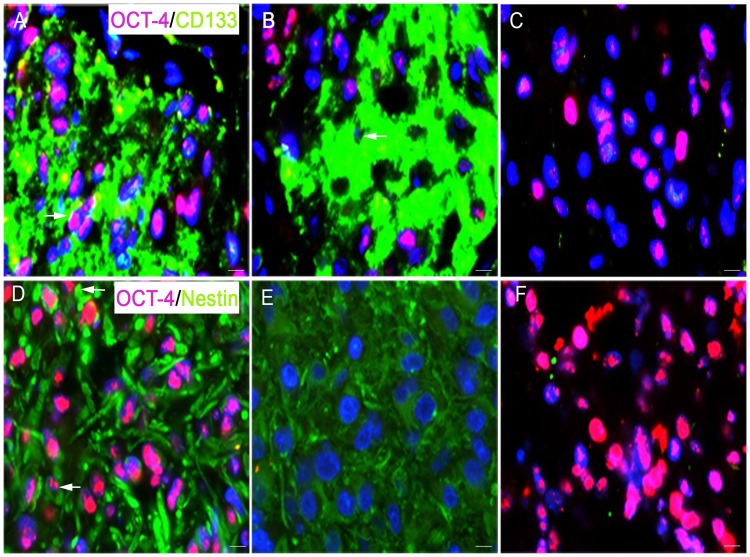
Double immunofluorescence staining with Oct-4 and the TSC markers CD133 and nestin. (A, B) The staining showed that CD133 positive (green) perivascular niches were found with both high (A, arrow) and low (B, arrow) Oct-4 (red) expression levels. (C) Areas were also found with Oct-4 positive dispersed single cells without CD133 staining. (D, E) When Oct-4 (red) was combined with nestin (green), Oct-4 and nestin were found to be co-expressed in some areas (D, arrows), but areas were also identified with only nestin positive cells (E) or only Oct-4 positive cells (F). Nuclei were counterstained with DAPI (blue). Scale bar indicates 50 μm, 400x.

Double immunofluorescence stainings with Oct-4/CD133 and Oct-4/nestin was performed to identify potential co-expression of stem cell markers. Double immunofluorescence staining with Oct-4/CD133 revealed that CD133 positive perivascular niches were found with both high (Fig 5A, arrow) and low (Fig 5B, arrow) Oct-4 expression levels. Areas were also found with Oct-4 positive dispersed single cells without CD133 staining (Fig 5C). Oct-4/nestin double immunofluorescence staining showed that Oct-4 and nestin were co-expressed in some areas (Fig 5D, arrows). There were also nestin positive areas without Oct-4 staining (Fig 5E), and Oct-4 positive dispersed single cells without nestin staining (Fig 5F).

## Discussion

In this study, Oct-4 was expressed in all tumors, and we observed a grade-dependent increase in expression, with the highest expression in GBMs. However, no association was found between Oct-4 protein expression and overall survival.

We have recently demonstrated that Oct-4 is expressed in human astrocytic brain tumors [18] as also found in the present study. In the current study we both identified Oct-4 expression in the nucleus as well as a weak cytoplasmic staining of most astrocytic brain tumors. Du et al. found expression of Oct-4 in the nuclei, but not in the cytoplasm of glioma cells [16]. However, cytoplasmic staining has been observed in other cancers such as oral cancer [28]. The detection of both nuclear and cytoplasmic staining in our study may be explained by a sensitive staining and is in line with Oct-4 been a transcription factor been synthesized in the cytoplasm followed by transportation to the nucleus.

Furthermore, we demonstrated a significant increase in Oct-4 tumor cell fraction with increasing tumor grade. This is in accordance with TCGA mRNA results and with two other previous IHC studies by Du et al.[16] and Elsir et al.[17]. Du et al. found that the fraction of Oct-4 increased in a grade-dependent manner [16]. However, these results were based on scoring of 41 different gliomas comprising 9 DAs, 3 oligodendrogliomas, 2 ependymonas, 5 AAs, 1 anaplastic ependymona, 2 anaplastic oligodendrogliomas and 19 GBMs. In another recent IHC study using tissue microarrays Elsir et al. showed that Oct-4 fraction among other TSC-related markers were up-regulated in 98 high-grade gliomas compared with 80 low-grade gliomas of oligodendroglial and astrocytic type [17]. However, in both studies semi-quantitative pathologist-based scoring systems was used and no statistical comparison was made. To provide a more unbiased quantification approach we used blinded quantitative stereology and systematic random sampling [29]. Moreover, we focused on and compared astrocytic tumors only. An unbalanced proportion of oligodendroglial and astrocytic tumors when comparing Oct-4 expression and grade may be a potential source of bias. Although this may influence the TCGA mRNA results and the IHC results by Du et al.[16] and Elsir et al.[17], an increase of Oct-4 with grade seems to be a robust finding. Similar results have been obtained in oral cancer [28] and urothelial cancer [30] suggesting an association of Oct-4 with tumor malignancy in cancer in general.

Neither the fraction nor the intensity of Oct-4 had significant prognostic impact when investigating all astrocytomas combined or when examining the individual astrocytoma grades. This is in line with the study by Elsir et al.[17]. In the TCGA dataset mRNA Oct-4 expression was associated with shorter overall survival when all grades were combined, but not in GBMs. This may be true, but could also represent bias due to an unbalanced proportion of oligodendroglial and astrocytic tumors in the two arms of the survival analysis. On the other hand, our IHC results may by biased by a saturated Oct-4 staining not showing dynamic staining intensities of Oct-4. This may also explain why Oct-4 intensity is similar for individual grades. However, we found an interesting correlation between Oct-4 staining intensity and mIDH1in AAs with a significantly higher intensity in mIDH1 compared to wtIDH1. High Oct-4 levels, especially high intensity, tended to predict a shorter patient survival, although the number of patients included in the analysis was limited and further investigation is required.

Using double immunofluorescence we found that CD133^+^ positive perivascular niches both contained CD133 positive cells with and without co-expression of Oct-4. Nestin, another TSC-related marker was also found to be co-expressed with Oct-4. This may imply that the expression of TSC markers in GBMs is not directly related and that these stem cell proteins may be active at different levels in the differentiation hierarchy. CD133 has previously been described by us [4] and others [2] to identify perivascular niches. The CD133 positive tumor cells are proposed to be involved in radio- and chemoresistance [3] and the detected co-expression with Oct-4 may suggest that Oct-4 also play a role in maintaining this therapeutically resistant type of glioma cells.

In conclusion, high Oct-4 mRNA expression was associated with grade and short survival in the TCGA dataset. Oct-4 expression levels in 114 astrocytic brain tumors assessed by IHC staining increased with WHO tumor grade but seemed to be without independent prognostic potential in GBMs. In the AA subgroup, Oct-4 intensity was associated with IDH1 status. However, identification of a potential prognostic value for Oct-4 in AAs requires additional studies using larger patient cohorts.
